# Psychiatric Comorbidities in Hyperacusis and Misophonia: A Systematic Review Protocol

**DOI:** 10.1192/j.eurpsy.2023.2196

**Published:** 2023-07-19

**Authors:** A. L. M. Rodrigues, A. R. Ferreira, H. Aazh, L. Fernandes

**Affiliations:** 1 Faculty of Medicine; 2 Department of Clinical Neurosciences and Mental Health, University of Porto, Porto, Portugal; 3 Audiology, Royal Surrey County Hospital, Guildford, United Kingdom; 4 Psychiatry Service, Centro Hospitalar Universitário de São João, Porto, Portugal

## Abstract

**Introduction:**

Decreased sound tolerance amongst individuals can be divided into two conditions: Hyperacusis and Misophonia. Hyperacusis is the perception of certain everyday sounds as too loud or painful. Misophonia is characterized by heightened emotional reaction to a sound with a specific pattern and/or meaning to an individual, with the context in which occurs being relevant. Scattered evidence from clinical research suggests that Hyperacusis and Misophonia can co-occur with a wide range of psychiatric disorders. These factors can have an impact on the severity of the symptoms and subsequently, in the clinical management of these patients. A better understanding these comorbid conditions is important as it could help to clarify its underlying mechanisms and ultimately, to improve the care of these patients. Despite this, no attempt has been made to synthesize the spectrum of such co-occurring disorders.

**Objectives:**

To conduct a systematic review of the available evidence on the prevalence of psychiatric disorders in patients with Hyperacusis and Misophonia, and to explore which factors may influence prevalence estimates.

**Methods:**

Preferred Reporting Items for systematic Reviews and Meta-Analyses (PRISMA) and Meta-analyses of Observational Studies in Epidemiology (MOOSE) recommendations will be followed. The CoCoPop (Condition, Context and Population) framework was used to develop the review question. Pubmed, PsycINFO, Scopus and Web of Science electronic databases will be searched, as well as grey literature, using key-terms in accordance with the pre-established research question. Additional manual searches will also be conducted. Searches will be limited to human studies and no date, language or country origin restrictions will be applied. Outcomes of interest will be the occurrence of comorbid psychiatric disorders in patients with Hyperacusis and Misophonia that are reported according to validated assessment methods. Retrieved records will be screened for eligibility by two independent reviewers using a two-phase approach (title and abstracts screening and full-text review). The methodological quality of primary studies will be assessed using the Joanna Briggs Institute (JBI) – Critical Appraisal Tools, depending on study design, and data will be extracted independently using a standardized extraction form.

**Results:**

Quantitative data will be synthetized and presented in text and tabular format. Studies heterogeneity will be verified and if feasible, a meta-analysis will be conducted.

**Conclusions:**

It is expected that this systematic review will provide evidence of a significant prevalence of a wide range of psychiatric comorbidities in patients with Hyperacusis and Misophonia, supporting the importance of screening these patients for psychiatric disorders.

**Disclosure of Interest:**

None Declared

